# A Transcriptomic Comparison of Two Bambara Groundnut Landraces under Dehydration Stress

**DOI:** 10.3390/genes8040121

**Published:** 2017-04-18

**Authors:** Faraz Khan, Hui Hui Chai, Ishan Ajmera, Charlie Hodgman, Sean Mayes, Chungui Lu

**Affiliations:** 1 School of Biosciences, University of Nottingham, Sutton Bonington Campus, Nottingham LE12 5RD, UK; stxfk5@nottingham.ac.uk; 2 Crops for the Future, Jalan Broga, 43500 Semenyih, Selangor Darul Ehsan, Malaysia; huihui.chai@nottingham.edu.my; 3 Centre for Plant Integrative Biology, University of Nottingham, Sutton Bonington Campus, Nottingham LE12 5RD, UK; bhzia@exmail.nottingham.ac.uk (I.A.); charlie.hodgman@nottingham.ac.uk (C.H.); 4 School of Animal Rural and Environmental Sciences, Nottingham Trent University, Clifton Campus, Nottingham NG11 8NS, UK; chungui.lu@ntu.ac.uk

**Keywords:** Bambara groundnut, landraces, dehydration stress, cross-species microarray analysis

## Abstract

The ability to grow crops under low-water conditions is a significant advantage in relation to global food security. Bambara groundnut is an underutilised crop grown by subsistence farmers in Africa and is known to survive in regions of water deficit. This study focuses on the analysis of the transcriptomic changes in two bambara groundnut landraces in response to dehydration stress. A cross-species hybridisation approach based on the Soybean Affymetrix GeneChip array has been employed. The differential gene expression analysis of a water-limited treatment, however, showed that the two landraces responded with almost completely different sets of genes. Hence, both landraces with very similar genotypes (as assessed by the hybridisation of genomic DNA onto the Soybean Affymetrix GeneChip) showed contrasting transcriptional behaviour in response to dehydration stress. In addition, both genotypes showed a high expression of dehydration-associated genes, even under water-sufficient conditions. Several gene regulators were identified as potentially important. Some are already known, such as *WRKY40*, but others may also be considered, namely *PRR7*, *ATAUX2*-11, CONSTANS-like 1, *MYB60*, *AGL-83*, and a Zinc-finger protein. These data provide a basis for drought trait research in the bambara groundnut, which will facilitate functional genomics studies. An analysis of this dataset has identified that both genotypes appear to be in a dehydration-ready state, even in the absence of dehydration stress, and may have adapted in different ways to achieve drought resistance. This will help in understanding the mechanisms underlying the ability of crops to produce viable yields under drought conditions. In addition, cross-species hybridisation to the soybean microarray has been shown to be informative for investigating the bambara groundnut transcriptome.

## 1. Introduction

Dehydration is one of the major stresses that inhibits plant growth and can reduce crop productivity. Hence, drought resistance is a key target in helping to ensure global food supply. Plants respond to dehydration stress in three broad approaches: (1) Dehydration escape; (2) Dehydration avoidance; and (3) Dehydration tolerance. Such mechanisms are seen in a range of leguminous species, including the mung bean [1] and pigeon pea [2]. Dehydration escape is the ability of plants to complete their growth cycle and reach maturity with successful reproduction before the shortage of water reaches damaging levels [3]. Mechanisms of avoidance include improved root traits for a greater extraction of soil moisture, stomatal closure, a decreased radiation absorption through leaf rolling, a decreased leaf area for reduced water loss, and the accumulation of osmoprotectants such as proline, trehalose, and dehydrins [4]. Dehydration tolerance allows plants to survive through improved water-use efficiency, i.e., performing all of the biological, molecular, and cellular functions with minimal water. Numerous studies on the effects of dehydration stress on staple crops have been reported [1,2,4,5,6,7,8,9,10].

Reduced water availability causes the production of abscisic acid (ABA), the phyto-hormone which initiates stomatal closure and influences other aspects of plant growth and physiology. It is responsible for regulating a broad range of genes during the dehydration response. The SNF1-related protein kinase, AREB (ABA-responsive element)/ABF are the key regulators of ABA signalling [11]. Improving the dehydration tolerance has also been linked to a reduction in shoot growth, while root growth is maintained, leading to an altered partition between the root and shoot. This process is achieved by cell-wall synthesis and remodelling. The formation of reactive oxygen species (ROS) and lignin peroxidases are the key steps involved in cell wall thickening.

Stomatal closure limits the CO_2_ uptake by leaves, which leads to a reduction in photosynthesis as the leaf’s internal CO_2_ is depleted. Severe dehydration stress also limits photosynthesis by down-regulating the expression of ribulose-1, 5-bisphosphate carboxylase/oxygenase (Rubisco), fructose-1,6-bisphosphatase (FBPase), phosphoenolpyruvate carboxylase (PEPCase), pyruvate orthophosphate dikinase (PPDK), and NADP-malic enzyme (NADP-ME) [12]. Plant responses to dehydration affect vegetative growth by reducing the leaf-area expansion and total dry matter, which in turn decreases light interception [13]. Under dehydration stress, wheat (*Triticum dicoccoides*) shows a reduction in the number of grains, grain yield, shoot dry weight, and harvest index [8]. In soybean specimens (*Glycine max*), the loss of seed yield was reported to be greatest when dehydration appeared during anthesis and the early reproductive stages [6,7,8,9].

A range of dehydration stress-related genes have been identified in *Arabidopsis thaliana*, rice (*Oryza sativa*), and other model plants [14]. These can be classified into two main groups: (i) Effector proteins, whose role is to alleviate the effect of the stress (such as water channel proteins, detoxification enzymes, LEA proteins, chaperones, and osmoprotectants); and (ii) Regulatory proteins, which alter the expression or activity of effector genes and modify plant growth, such as the transcription factors DREB2 and AREB, and also protein kinases and phosphatases [15].

In recent years, plant breeders have turned to landraces (i.e., locally adapted genetically mixed populations) for trait improvement in various crops, including barley [16], sorghum [17], sesame [18], and soybean [19]. An early attempt to investigate the use of landraces in addressing the problem of dehydration tolerance has been carried out in wheat [20], although this did not delve into the specific genetics conferring the desirable traits. An alternative approach to identifying the genes conferring dehydration avoidance and tolerance is to study species that are already resilient under arid conditions. In this regard, bambara groundnut (*Vigna subterranea* (L) Verdc.) is a potential candidate. It is an underutilised, drought-resistant African legume, which is mainly grown in sub-Saharan Africa [5,6,7,8,9,10,11,12,13,14,15,16,17,18,19,20,21] and is sometimes used as an intercrop with major cereals, such as maize, because of its nitrogen fixing potential [22]. Bambara groundnut is considered as a drought resistant crop with a reasonable protein content (18% to 22%), a high carbohydrate content (65%), and some level of lipids (6.5%) [23]**,** with a similar overall composition to chickpea. A number of bambara groundnut landraces have well-developed tap roots which grow up to a height of 30–35 cm [24].

From the results of Mabhaudhi et al. [25], bambara groundnut has been shown to adopt dehydration-escape mechanisms, including a shortened vegetative growth period, early flowering, a reduced duration of the reproductive stage, and early maturity under dehydration stress. Such responses are likely to be employed where the initial plant growth is based on stored soil water, but further rain is unlikely. It has been reported that bambara groundnut responds to dehydration stress by partitioning more assimilate into the root, relative to the shoots, so that a greater soil volume can be exploited [26,27]. Nyamudeza [27] also observed that bambara groundnut allocated a greater fraction of its total dry weight to the roots than the groundnut, irrespective of the available soil moisture. This would suggest that bambara groundnut commits a greater supply of assimilates to root growth, irrespective of the soil moisture status. This strategy may have clear advantages when water subsequently becomes limited, but there could be a trade-off with the yield under benign environments. A greater root dry-weight was also reported when the bambara landrace, Burkina, was subjected to dehydration stress [28]. Dehydration-avoidance traits have also been observed, especially the accumulation of proline [21] and a reduced leaf area [29].

This study aims to investigate the effects of dehydration on gene expression in this reportedly drought-resistant species. The transcriptomes of two genotypes (DipC and Tiga Nicuru (TN)) were sampled, to identify what is common and how they differ in their response to a prolonged, but slowly intensifying, dehydration treatment. The climatic conditions in their native regions (Botswana and Mali, respectively) suggest that they are likely to have evolved in regions which would select for drought resistance, while potentially exhibiting some variation in the mechanisms employed to deal with dehydration, as they are morphologically and phenologically distinct [30]. Chai et al. [30,31] reported that transgressive segregation was observed in the segregating F_5_ population derived from the TNxDipC cross. The contrast between the two parental lines for a number of traits such as the days-to-maturity, stomatal conductance, 100-seed weight, leaf area, internode length, peduncle length, pod number per plant, and leaf carbon (delta C^13^) isotope analysis, suggest that some of these mechanisms for adaptation to dehydration could be non-identical in the two genotypes. For example, delta C^13^ was associated with a higher yield as observed in DipC, compared to TN [30]. In addition, the results showed that there were lines in the segregating population that performed better in terms of the ability to produce higher yields under drought conditions than the parental genotypes. Hence, evaluating the transcriptome of the two parental lines under dehydration stress could be a good indicator to investigate the molecular mechanism occurring in the two genotypes and its relationship to phenology and phenotype.

As a complete genome sequence is not available and microarray tools are still to be developed in this species, cross-species hybridisation with the Affymetrix Glycine-max microarray was investigated to test if this approach is acceptable for bambara groundnut transcriptomics, as it has been successful for other species [32,33,34].

## 2. Materials and Methods

### 2.1. Plant Materials

In this study, the experiment was conducted in the FutureCrop controlled tropical glasshouses at the School of Biosciences, Sutton Bonington Campus, University of Nottingham, UK. Two genotypes of bambara groundnut, DipC and TN, were planted in both ‘Water-limited’ and ‘Water-sufficient’ control plots.

### 2.2. Site Descriptions and Experimental Design

Plants were grown over a period of five months. A 12-hour photoperiod was created using an automated blackout system (Cambridge Glasshouses, Newport, UK), with day and night temperatures set at 28 °C and 23 °C respectively. Trickle tape irrigation with PVC micro-porous tubing was placed beside each plant row. The plants were irrigated at 06:00 h and 18:00 h for 20 min, with a measured flow rate of 1 L/h per tube, and each tube was 5 m in length. Two independent soil pits (5 m × 5 m × 1 m) containing sandy loam soil were used in the glasshouses. These were isolated from the surrounding soil by a Butyl liner and concrete pit structure with gravel drainage for separate water-limited and water-sufficient plots. The PR2 water profile probe (Delta-T devices, Cambridge, UK) was used to measure the soil moisture content. A randomised block design (RBD) with three blocks for each soil pit was implemented for this experiment. Three replicate plants for the water-sufficient plot (continuously irrigated) and four replicates for the water-limited treatment plot were used. Three seeds were sown per replicate at a depth of 3–4 cm with a spacing of 25 cm × 25 cm between each final plant position and multiple plants were later thinned to one plant per replicate at 20 days after sowing (DAS). Figure S1 shows the treatment regime. The irrigation system for the water limited treatment plot was turned off at 50 DAS and resumed at 92 DAS for plant recovery (in total, six weeks of treatment after 100% flowering). Normal irrigation continued for the water-sufficient plot throughout. The water-limited treatment was continued until an average of a 50% reduction in *stomatal conductance* was observed. Leaves from water-sufficient and water-limited plants were collected at 92 DAS before recommencing irrigation, while those from ‘recovered’ plants were collected at 107 DAS after watering was resumed at 92 DAS. Labelled aluminium foil was used to wrap the harvested leaves, which was then transferred into liquid nitrogen for long term storage. All samples were stored in a −80 °C freezer before RNA extraction. DNA extraction from the two parental genotypes was completed using the DNA extraction Qiagen kit handbook.

### 2.3. RNA Extraction

RNA was extracted using the RNeasy Qiagen kit (Qiagen, Manchester, UK), according to the manufacturer’s instructions. DNA was eliminated using DNase. A total of 80 µL of DNase I incubation mix, containing 10 µL DNase I stock solution and 70 µL buffer RDD, was added and incubated at room temperature for 15 min. Nanodrop readings and gel electrophoresis were performed to check the quality and quantity of RNA, as RNA samples required 100 ng/µL for 10 µL for microarray analysis. To make sure that the samples were free from active RNAse, 0.63 µL of 40 U/µL RNasin (Promega, Southampton, UK) was added for every 25 µL of the RNA sample. All samples were tested on an Nanodrop and Agilent bioanalyser for integrity (looking at the quality (ratio of 2.0) and integrity (a ratio of 2 for 28S/18S) for respective quantitation) before preparation for the microarray.

### 2.4. cRNA and Genomic DNA Affymetrix Labelling and Hybridisation

The above RNA extracts were reverse transcribed to synthesize double stranded complementary DNA (cDNA). After purification of the double-stranded cDNA products, the sample was transcribed in vitro to generate Biotinylated complementary RNAs (cRNAs), followed by purification and fragmentation. The purified and fragmented cRNAs were then hybridised to the Affymetrix Soybean Gene Chip array (ThermoFisher Scientific, Lutterworth, UK). The scanned arrays produced CEL raw data files that were loaded onto Genespring GX version 13.1 (Agilent Genomics, Santa Clara, CA, USA) for further analysis. The extraction of genomic DNA (gDNA) from the two genotypes was performed using the DNA extraction Qiagen kit according to manufacturer’s instructions. Extracted DNA was labelled and hybridised to the Affymetrix Soybean TEST3 array and resulted in the generation of gDNA cell-intensity files (CEL files), after scanning. To identify probe pairs that efficiently hybridise to the gDNA, a series of user defined threshold values were evaluated for the signal intensity. The perfect match (PM) probes were selected for interpreting the GeneChip arrays challenged with RNA from the species of interest [35].

### 2.5. Probe Selection and Identification of Differentially Expressed Genes

The soybean array contained 37,500 probe sets, each containing 11 probe pairs per probe-set. For each genotype, custom CDF files were obtained, with reference to their gDNA hybridisation signal strength [36] for a subsequent estimation of the transcript levels. RNA CEL files were normalised in GeneSpring [37] using the Robust Multi-array Average. Differentially expressed genes (DEGs) were calculated using a *t*-test test (corrected by Benjamin Hochberg false discovery rate (FDR) multiple testing). Probe-sets with a FDR corrected *p*-value ≤ 0.05 and fold change of >2 were considered to be differentially expressed (either up or down regulated). Principal Component Analysis (PCA) was also carried out in GeneSpring and Bioconductor package “prcomp”. BINGO was used for discovering (from input gene lists) over-represented terms from the Gene Ontology [38].

### 2.6. Construction of the Co-Expression Network

Co-expression network analysis was carried out using the DeGNserver [39] and cytoscape 3.4 [40]. Separate networks were generated for each genotype. The input probe-sets were restricted to those that were differentially expressed between each pair of treatments (water-limited, water-sufficient and recovery) and RMA (Robust Multi-Array Average)-normalised values were used across all samples. Links were assigned between pairs of nodes (i.e., probe-sets) when their Spearman’s Rank correlation was 0.9. The co-expression network was imported into cytoscape for visual representation and network analysis. For each genotype, another input file was made which, for each probe-set, defined the parent (DipC or TN), the direction of differential expression caused by dehydration (up or down), and the role identified through homology in relation to drought resistance. This aided the interpretation of the combined network derived from both genotypes.

### 2.7. Expression Validation of Differentially Expressed Genes Using Real-Time qPCR

Four genes which were potential candidate dehydration-associated genes (based on their functional annotations) with a differential expression level of >2-fold change and FDR corrected *p*-value ≤ 0.05 from the differential expression analysis, were chosen for quantitative PCR (qPCR) validation. The actin-11 from the available bambara groundnut transcriptome sequence was used as a housekeeping gene. The actin-11 gene is known to be one of the most stable reference genes for gene expression normalisation and has been used in soybean and rice specimens [41,42]. PCR forward and reverse primers were designed using Primer-BLAST [43] for the chosen genes. The primers were designed in three steps. Firstly, the target gene sequence to which the primers needed to be designed was downloaded from the soybean database. Secondly, the soybean-specific target gene sequence was blasted against a bambara groundnut transcriptome generated from RNA-sequencing data for a low-temperature stress experiment [44], by creating a BLAST database. Thirdly, the target gene sequence obtained from the bambara groundnut BLAST database was used to search through the BLAST database at NCBI to add weight to the selection of this sequence. Once the gene sequence was identified in the BLAST database, it was utilised to design primers with an appropriate primer size, GC content, and melting temperature (Tm) using Primer-BLAST. PCR was performed to check the quality of all the primers designed for the four dehydration-associated genes. PCR analysis was performed using the 7000 Sequence Detection System (Applied Biosystems, Cheshire, UK). The annealing temperature was set to 60 °C for the primer designed for the genes for *PAL1* (Phenylalanine ammonia-lyase 1) and *COMT* (3-Caffeic acid o methyltransferase), and 58 °C for the Beta-fructofuranosidase and *UBC-2* (ubiquitin conjugating enzyme-2) genes. The cycling parameters were set as: 95 °C for 10 min, 40 cycles of denaturing at 95 °C for 30 s, annealing at 60 °C/58 °C for 30 s, and extension at 72 °C for 30 s. First strand cDNA synthesis for all the RNA samples was carried out using a SuperScript III First-Strand Synthesis kit (ThermoFisher Scientific, Lutterworth, UK). The first-strand cDNA was prepared for analysis by qPCR using PerfeCta SYBR Green SuperMix (Quantabio, Beverly, MA, USA) containing 2X reaction buffer (with optimized concentrations of MgCl_2_), dNTPs (dATP, dCTP, dGTP, dTTP), AccuStart Tag DNA Polymerase (Quantabio, Beverly, MA, USA) SYBR Green 1 dye, and stabilizers. The synthesized cDNA was cleaned from the remaining RNA using the enzyme mix included in the kit (*Escherichia coli* RNase H). The qPCR components were prepared for 10 µL reactions and Melt-curve analysis was performed. The sample cycle threshold (Ct) was standardized for each template based on the actin-11 gene control amplicon behaviour. The 2^−ΔΔCt^ method was used to analyse the relative changes in gene expression from the qRT-PCR experiment [45]. To validate whether the right PCR product was generated for the expression studies, the desired fragment of intact cDNA for all genes was sent for sequencing after the gel extraction using a QIAquick Gel Extraction Kit (Qiagen, Manchester, UK).

## 3. Results

### 3.1. Probe Selection Based on gDNA

**Figure 1:**
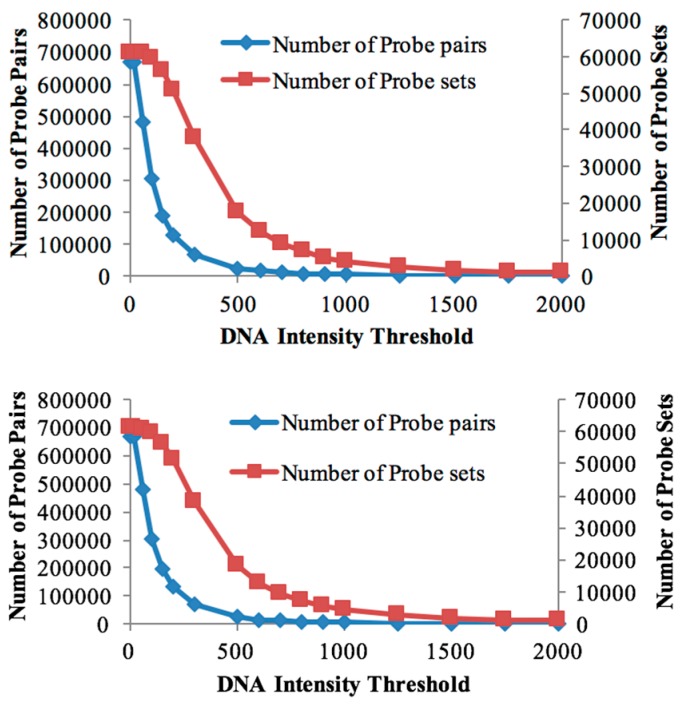
Effect of intensity thresholds. Number of probe pairs (blue line) and probe sets (magenta line) retained for DipC (**top**) and Tiga Nicuru (TN) (**bottom**) respectively at different genomic DNA (gDNA) intensity thresholds.

The genomic DNA of both genotypes was individually hybridised to the Affymetrix Soybean GeneChip array to study the global genome hybridisation for probe selection. The numbers of retained probe-pairs and probe-sets are shown in Table 1. With increasing threshold values, the number of probe-pairs retained in the probe mask file started decreasing rapidly (Figure 1), while the number of probe-sets (representing genes) decreased at a slower rate. This suggests that, even at higher gDNA hybridisation thresholds, at least some of the gene-designed oligonucleotides are cross-hybridising for many of the probe-sets and that the cross-species array approach is a reasonable approach for bambara groundnut transcriptomics.

The number of retained probe-sets and probe-pairs on the Soybean chip for both the DipC and TN gDNA hybridisations were determined, corresponding to each threshold value (Table 1). A custom CDF file with a threshold of 100 was chosen for differential expression analysis in both genotypes, as it allowed for a good sensitivity to detect the maximum number of differentially-expressed transcripts (Table 1). Furthermore, both genotypes were found to be highly similar in terms of the probe-sets detected at this threshold. A total of 59,533 probe-sets were common to both genotypes at the threshold of 100, while 249 and 302 probe-sets were specific to DipC and TN, respectively. These results therefore suggest a high sequence similarity (>99%) at this level of sequence sampling.

### 3.2. Principal Component Analysis

**Figure 2:**
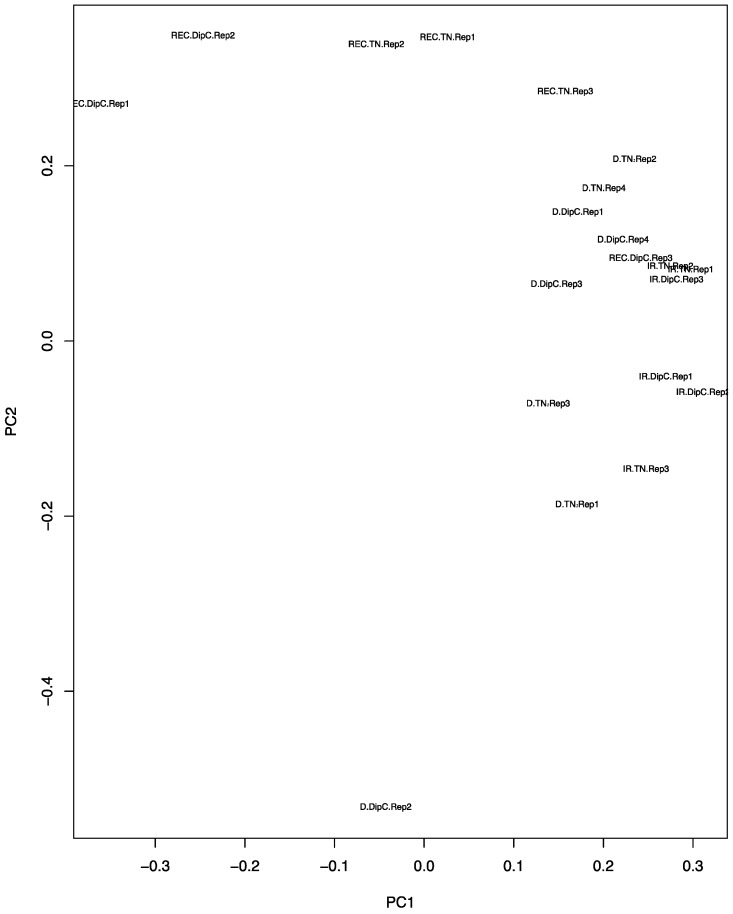
Principal component analysis (PCA) plot of the expression data from the microarrays. The principal components PC1 and PC2 values for each chip have been placed on a scatter plot. Each chip result is defined by a three-part character string consisting of the treatment, genotype, and replicate number. IR, D, and REC refer to water-sufficient, water-limited, and recovery treatment, respectively; the genotypes are DipC and TN; and Rep1–4 refers to the specific biological replicate. Note, the water-sufficient and recovery treatments have only three replicates, while dehydration has four.

The PCA plot (Figure 2) shows that, under water-sufficient treatment, the two genotypes appear to have similar transcriptomes. The first two Principal Components account for 25.45% and 17.11% of the variance, respectively, suggesting that it is due to a range of hybridisation/expression differences between the chips. Recovery after dehydration stress, however, caused the most variation and suggests that the recovery transcriptome does not return to the water-sufficient state (control). The DipC water-limited treatment sample ‘D.DipC.Rep2’ could be a potential outlier and this needs to be borne in mind in further analysis. The 3D PCA plots of genotype-specific data showed a good separation of the three treatments (conditions) and better PCA scores (see Figure S2).

### 3.3. Gene Expression Under Water-Sufficient Conditions

It is pertinent to consider the state of the genotype transcriptomes before any dehydration treatment has taken place. However, owing to the high background noise in microarray studies, it is unclear what intensity level defines a gene as being transcribed. Figure S3 shows that the ranked intensity values follow a roughly sigmoidal curve. The point of inflection (at which the declining gradient is at its shallowest) covers the top two-thirds of the probe-sets, and corresponds to an RMA value of 0.97. This may be a stringent cut-off, given that an RMA value of one corresponds to the average across all probe-sets on the array, but it ensures that there were few, if any, false positives. This left 39,855 probe-sets for DipC and 39,890 for TN. There are 26,496 probe-sets in common between the two genotypes, suggesting differences in the general transcriptional behaviour of the two genotypes.

Each genotype had a little over 90 probe sets with functional annotations related to ABA signalling and dehydration responses (see Tables S1 and S2), of which 60 were common to both. These include homologues of much of the ABA synthesis and response network, the DREB1 transcription factor, Early-Response to Dehydration proteins 3, 4, 8, 14–16, and 18, four osmoprotectant genes, two dehydration-response genes influencing photosynthesis, and 21 other probe sets corresponding to dehydration-associated proteins of an unknown function (see Table S3). Table S4 lists the genes differentially expressed between the two water-sufficient treated genotypes, but at this stage, nothing stands out as remarkable.

### 3.4. Identification of Differentially Expressed Genes

For DipC and TN, the numbers of genes differentially expressed as a result of the dehydration and recovery treatments, and detected by the cross-species microarray approach, are shown in Table 2, with the full lists of probe-sets and functional annotations presented in Tables S5–S8. The top upregulated and downregulated genes in DipC and TN are shown in Table 3 and Table 4, respectively. The numbers for DipC were consistently higher than for TN, and the water-limited treatment caused more down- than upregulation, while recovery had the reverse effect.

Recovery led to many more differentially expressed genes (486 and 391) than dehydration stress (189 and 81). There were six possible system effects that can be gleaned from these data (Figure S4). The upregulated genes under the water-limited treatment that returned to a water-sufficient state on recovery and the downregulated genes that returned to a normal expression at recovery are the strictly dehydration-responsive genes (~75% in both genotypes), while those that significantly changed and did not return to the pretreatment levels (~25%) correspond to a dehydration-induced state change. The latter may be due to epigenetic effects, such as a change in the methylation state of gene-regulatory regions. The larger numbers of differentially expressed genes from water-limited conditions to recovery may be accounted for by aging and other highly variable factors (see Figure 2), such as the soil conditions in each pit.

The fold changes of the upregulated genes under dehydration stress in both genotypes are relatively small (mostly < 4-fold). Furthermore, there were only nine differentially expressed genes which were common to both genotypes (see Table 5). The only common upregulated gene was beta-fructofuranosidase, which hydrolyses sucrose to provide more glucose, hence playing a potential role in osmoprotection and energy production. In contrast, half of the common downregulated genes were related to transcription and also play roles in stomatal regulation. Excluding the potential outlier, ‘D.DipC.Rep2’ had little effect upon the common gene analysis (Table S9), so it has been included in subsequent analyses.

Mostly, the upregulated genes under dehydration stress in DipC relate to the secondary metabolism of cell-wall components, while the TN genes include transcription-related factors, most notably a CONSTANS-like gene. Furthermore, GO term overrepresentation analysis for both DipC and TN showed an emphasis on various metabolic processes related to cellular amino acids and their derivatives, secondary metabolites and carbohydrates (Table S10). Hence, despite the genomic hybridisation mask demonstrating that the pure hybridisation was very similar between the two genotypes, there is a very different transcriptional response to dehydration stress by each genotype. Microarray data has a limited dynamic range, even when within species, so it is important to validate a small set of microarray observations. Hence, validation through qRT-PCR was performed.

### 3.5. Confirmation of Candidate Dehydration-Associated Genes by Real-Time qRT-PCR

**Figure 3:**
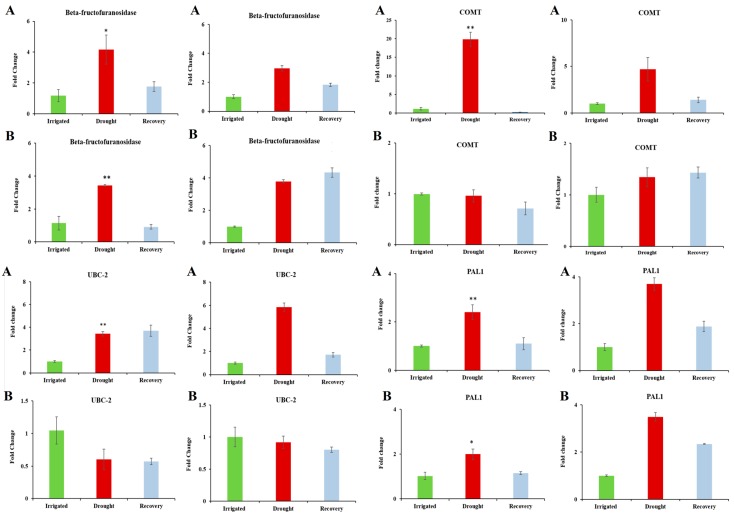
Comparison of qPCR and microarray intensity values: Rows (**A**) and (**B**) respectively refer to results for DipC and TN. The left- and right-hand pairs of columns correspond to the qPCR and microarray values for DipC and TN, respectively. The gene under study is named at the top of each panel. In order, the investigated genes are Beta-fructofuranosidase, *COMT*, *UBC-2*, and *PAL1*. qPCR and Microarray values are shown as fold changes with respect to the water-sufficient treatment (Irrigated). Error bars denote the standard error. Single and double asterisks indicate that *p*-value is less than 0.05 and 0.01, respectively, which was assessed by the paired *t*-test between groups. Irrigated and Drought refer to water-sufficient and water-limited treatments, respectively.

Four differentially expressed genes (*PAL1*, Beta-fructofuranosidase, *COMT* and *UBC-2*) were chosen for further analysis, as they showed high levels of expression under water-limited treatment [46,47,48,49,50,51,52,53,57,58,59,60] (Table 5 and Table 3) and are dehydration-associated genes based on their functional annotations. Figure 3 shows the results of qPCR analysis. The transcript levels of Beta-fructofuranosidase, *COMT*, and *UBC-2* confirmed the expression trends seen in the microarray data. *PAL1* showed the expected increase in DipC, and an increase in TN was observed, which was not observed in the microarray results. The reason for this is unclear.

### 3.6. Transcription Factors Associated with Dehydration Stress

The DEGs genes from both genotypes identified various transcription-related factors (TFs). Common to both genotypes are the downregulation of *BRH1*, an *MYB*, *MEE59*, and *JMJD5*. The latter is a histone demethylase, so could suggest changes at the epigenetic level of gene expression. Its downregulation could result in the indirect repression of multiple genes. On top of the common genes, DipC shows the upregulation of two TFs (*WRKY51* and a *bHLH* TF) and the downregulation of four others (*ATAUX2-11*, *WRKY40*, a C2H2 Zn-finger, and three probe-sets for GIGANTEA). TN, on the other hand, shows the upregulation of genes for CONSTANS1-like, S1FA DNA-binding, and a double-strand RNA-binding protein (which can aid microRNA-mediated RNA degradation). The downregulated TFs in TN are *MYB60* and a second *MEE59*.

Co-expression networks were individually built for DipC and TN (see Tables S11 and S12), and the dehydration-specific network of each were merged. This resulted in more TFs being included, which are features of recovery treatment. By looking at the number of links that each node has in the genotype-specific and merged networks, it is possible to rank the potential importance of the different TFs (see Table 6). The DipC TFs had a higher number of links than TN. In the case of DipC, *WRKY40* stands out as being the TF with the most co-expressed genes, with *ATAUX2-11*, *PRR7*, and a Zinc-finger protein (GmaAffx.33796.3.S1_at) also looking relevant. For TN, however, the TFs are not ranked so highly, with CONSTANS-like 1 and *MYB60* showing the greatest involvement. For this genotype, the differentially expressed TFs in common with DipC seem almost as important.

## 4. Discussion

Landraces are a potentially valuable resource for finding genes conferring useful agricultural and processing traits. Bambara groundnut is an underutilised African legume whose landraces are adapted, in many cases, to arid conditions. We have developed single genotypes derived from landraces for analysis. There have been several dehydration studies carried out on bambara groundnut, but the molecular mechanisms of how the crop responds and adapts to dehydration stress are still under investigation. This study has carried out transcriptomic comparisons in two genotypes of bambara groundnut, DipC and TN, in an attempt to identify potential genes conferring advantageous traits for crop growth and yields in marginal environments.

Cross-species hybridisation to the soybean microarray has been shown to be informative for investigating the bambara groundnut transcriptome, as good gene (probe-set) retention was observed at high gDNA hybridisation thresholds. In support of the results, Bonthala et al. [44], reported a high correlation between cross-species microarray data and RNA-sequencing approaches for detecting differentially expressed genes under a cold temperature stress experiment in bambara groundnut. Probe-sets retained by the mask after genomic hybridisation are almost identical (>99%), suggesting that, at this level of resolution, the two genotypes are highly similar at the sequence level. Four known dehydration-associated genes, seen to be differentially expressed in these data, were subjected to qPCR, and supported the notion that the observed trends in the microarray data are valid.

The 26,496 probe sets common between the two genotypes, under irrigated conditions, (with a RMA cut-off of 0.97), include some sixty dehydration- and ABA-related genes. The latter include genes for producing osmoprotectants. They might provide two components of the dehydration avoidance capability of these genotypes, by retaining normal cell functioning when water access becomes limited. Clearly, if the plant has already activated part of the dehydration response, it could have multiple effects. The presence of osmoprotectants might draw in even more water than otherwise might be the case, and there will be a greater proportion of biomass devoted to root growth, resulting in even deeper roots that are better able to survive dehydration later on. Bambara groundnut is known to allocate a greater fraction of its dry weight to the roots than to the shoots, irrespective of the soil moisture status [27]. This strategy may have clear advantages when water subsequently becomes limited, suggesting an adaptation to harsh environments and a decision to prioritise survival. In addition, as bambara groundnut is grown in harsh environments and has not undergone intensive breeding for the yield and above ground biomass, this suggests that it still allocates more effort to developing root architecture to handle dehydration when it happens. Moreover, Nayamudeza [27] also stated that the fraction of total dry weight allocated to the roots in bambara groundnut is greater than that allocated to the groundnut. In addition, a relatively higher expression of dehydration-associated genes in both genotypes under water-sufficient treatment including *ABI1* (ABA Insensitive 1), *ABF1* (ABRE binding factor 1), *ERD4* (Early responsive to dehydration 4), and *RD19* (Response to dehydration 19), compared to other species such as Soybean [76] (see Figure S5), suggest that bambara groundnut could at least be in a partially ready state for dehydration, even in the absence of dehydration stress. However, further research is needed to validate this hypothesis.

Given that 59,782 and 59,835 probe-sets were used to evaluate the transcriptome changes after probe-masking in DipC and TN, respectively, there were only very small numbers of genes significantly differentially expressed (189 in DipC and 81 in TN) under water-limited treatment. It could be speculated that the slow and progressive dehydration stress might not cause significant shock to the plants.

The upregulated genes in both genotypes were subdivided into ~75% dehydration responsive (with expression levels returning to normal after recovery) and ~25% dehydration perturbed (where the expression levels remained altered). In the case of downregulated genes, 80–85% of the expression levels returned to being comparable with the non-stressed state. The dehydration-perturbed expression levels might be caused by changes at the chromatin level, through DNA methylation or histone modification, and it is therefore interesting to note that a protein-lysine demethylase is repressed by dehydration.

**Figure 4:**
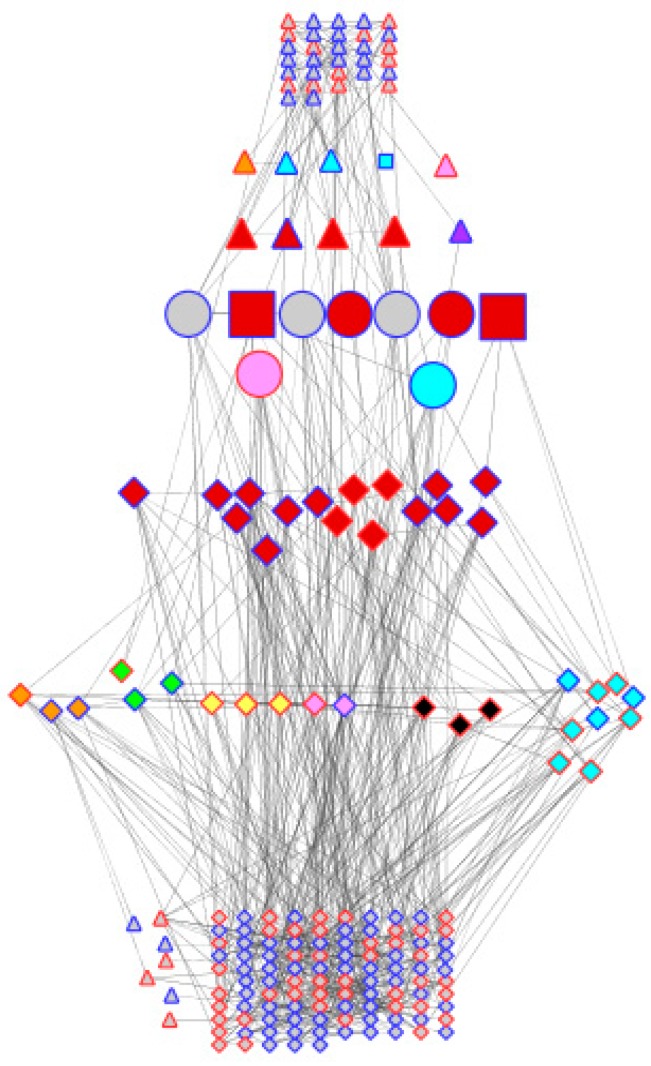
Comparison of genotype co-expression networks. Cytoscape has been used to layout the merged dehydration-responsive network of co-expressed probe-sets. Node shapes are triangles, diamond circles, and squares, respectively, for the differential expression of the probe-sets of TN, DipC, both (i.e., common), and both but affecting stomata. They have been coloured according to their activity in relation to the dehydration response: red (transcription), orange (cell wall), yellow (lignin synthesis), green (photosynthesis), blue (transporters), indigo (hormone signalling), pink (osmoprotection), black (oxidative stress), and grey (others). Node borders have been coloured red and blue to denote up- and downregulation under stress. Nodes have been arranged in seven horizontal bands with probe-sets in common in the middle flanked by TFs and hormone-signalling genes, other genes that play various roles in response to dehydration, and others. Nodes have been linked by the criteria of the co-expression analysis.

The above observations show that the two genotypes appear to be very similar in terms of their genotype (validating the comparability of the transcriptome data compared using the microarray), while exhibiting differences in their general transcriptional behaviour in water-sufficient conditions and in response to dehydration stress. However, when the sets of differentially expressed genes are compared, there is almost no overlap. Out of 189 and 91 genes differentially expressed in DipC and TN, respectively, only nine were common between the two genotypes, suggesting that some of the mechanisms for adaption to dehydration are substantially different in the two genotypes. Of these, Beta-fructofuranosidase contributes to osmoprotection [46,47], an MYB gene is associated with the stomatal opening in Arabidopsis thaliana [50], *BRH1* affects the stomatal density [51], and bsd2 affects photosynthesis in maize [52], while *JMJD5* plays an epigenetic role [49], as mentioned above. Figure 4 illustrates how two genotypes with very similar genomes may have adapted to achieve dehydration response traits (transcriptional and hormone signalling to affect cell-wall modification, lignin synthesis, photosynthesis, transporters, hormone signalling, osmoprotection, oxidative stress) through largely different sets of effector genes.

Several transcription factors that seem likely to play a role in the bambara groundnut dehydration response and which are common to both genotypes are *BRH1* and an MYB transcription factor, which are known to affect the stomata in *Arabidopsis thaliana* [50], and *JMJD5*. DipC shows a more significant response, with changes to *WRKY40*, and is of particular interest. It is a well-known member of plant dehydration-response networks [67] and is the most highly linked TF node in the co-expression networks. For DipC, the network also reveals the importance of *PRR7*, a core circadian clock component known to play a complex role in abiotic stresses [77]. It is somewhat surprising that TN does not show a >2-fold change in the expression of *WRKY40*, but it may have roles for CONSTANS-like 1 (another clock-related gene associated with flowering in rice that may be associated with abiotic stress in bambara groundnut [78]) and *MYB60*, which affect stomatal closure in *A. thaliana* [79], and *AGL-83*, a MADS-Box protein with an uncertain role.

## 5. Conclusions

Understanding the mechanisms underlying the ability of crops to produce viable yields under drought conditions is a priority for global food security. This study has examined the transcriptomic reponse to dehydration and recovery in two genotypes derived from landraces of bambara groundnut, in an attempt to investigate the molecular mechanisms occurring in the two landraces. In addition, this study also tested whether the cross-species hybridisation to the soybean microarray is suitable for investigating the bambara groundnut transcriptome. It was shown that many potential dehydration-responsive genes are expressed, even under water-sufficient conditions, in both landraces, suggesting that bambara groundnut could at least be in a partially ready state for dehydration, even in the absence of dehydration stress. In terms of differential expression, there were only a very small number of genes differentially expressed under water-limited treatment in both landraces, suggesting that the slow and progressive dehydration stress might not cause a significant shock to the plants. Although the transcription factors and dehydration-response genes were largely different between the two landraces, they may achieve the same effect in terms of survival under drought conditions. The DipC genotype displayed the differential expression of some well-known dehydration-associated transcriptions factors (especially *WRKY40*), while TN showed the differential expression of CONSTANS-LIKE 1 and *MYB60*. Cross-species hybridisation to the soybean microarray has been shown to be informative for investigating the bambara groundnut transcriptome, as good gene retention was observed at high gDNA hybridisation thresholds.

## Figures and Tables

**Table 1:** Retained probe-sets and probe-pairs at different threshold values.

Threshold Value	Number of Probe Sets (Soybean Chip Hyb. to DipC gDNA)	Number of Probe Sets (Soybean Chip Hyb. to TN gDNA)	Number of Probe Pairs (Soybean Chip Hyb. to DipC gDNA)	Number of Probe Pairs (Soybean Chip Hyb. to TN gDNA)	Number of DEGs in DipC	Number of DEGs in TN
20	61,072	61,072	670,388	670,388	6165	6165
60	60,877	60,895	479,538	482,352	6927	6814
**100**	**59,782**	**59,835**	**302,834**	**304,708**	**7183**	**7159**
150	56,266	56,511	190,570	193,522	7036	7159
200	51,071	51,319	129,806	132,521	6638	6731
300	37,813	38,000	66,907	68,106	5275	5345
500	17,469	18,176	23,464	24,693	2784	2911
600	12,258	12,930	15,701	16,650	2089	2170
700	8896	9566	11,193	12,061	1574	1673
800	6687	7208	8415	9070	1195	1291
900	5140	5657	6559	7140	958	1057
1000	4085	4482	5304	5733	802	877

gDNA: genomic DNA; DEG: differentially expressed gene; TN: Tiga Nicuru; Hyb: Hybridisation.

**Table 2:** Differentially expressed gene numbers.

	Water-Limited versus Water-Sufficient				Water-Limited versus Recovery	
	Up-Regulated under Dehydration	Down-Regulated under Recovery	Down-Regulated under Dehydration	Up-Regulated under Recovery	Up-Regulated	Down-Regulated
DipC	80	68	109	94	340	146
Tiga Nicuru	28	22	53	42	294	97

**Table 3:** Top upregulated genes in DipC and TN.

Gene Name	FDR	Fold Change	Gene Description	References
**UP-Regulated Genes in DipC**				
PAL1 (Phenylalanine ammonia-lyase 1)	0.018	3.901	Key enzyme involved in the biosynthesis of isoprenoid antioxidative and polyphenol compounds such as lignin and is involved in defense mechanism.	[53]
ATEP3/AtchitIV	0.001	3.845	Encodes an EP3 chitinase that is stimulated under abiotic stress.	[54]
TXR1(Thaxtomin A resistant 1)/ATPAM16	6.87 × 10^-5^	3.718	TXR1 is a component of a dispensable transport mechanism. Involved in negative regulation of defense responses by reducing reactive oxygen species (ROS).	[55]
Acetyl-CoA C-acyltransferase, putative / 3-ketoacyl-CoA thiolase	0.001	3.554	Functions in Jasmonic acid synthesis which plays a role in plant response to mechanical and abiotic stress.	[56]
UBC-2 (ubiquitin-conjugating enzyme 2)	0.004	3.407	Ubiquitination plays a part in increasing rate of the protein breakdown. Arabidopsis plants overexpressing UBC-2 were more tolerant to dehydration stress compared to the control plants.	[57]
Rho GDP dissociation inhibitor 2	0.001	3.348	Involves in the regulation of Rho protein and small GTPase mediated signal transduction.	[58]
Histidine amino acid transporter (LHT1)	0.001	3.256	Amino acid transmembrane transporter involved in apoplastic transport of amino acids in leaves.	[59]
COMT (3-Caffeic acid o methyltransferase)	0.006	3.234	Involved in lignin biosynthesis. High activation of lignifying enzymes was found in dehydration-stressed white clover (*Trifolium repens* L.), which lead to reduced forage growth.	[60]
Glycine decarboxylase complex H	0.005	3.113	Functions in photo respiratory carbon recovery. Carbon dioxide is found to be low in plants subjected to dehydration stress due to the closing of stomata in order to prevent water loss.	[61]
**Up-Regulated Genes in TN**				
Clp amino terminal domain-containing protein, putative	0.035	3.778	Protein and ATP binding.	
CONSTANS-LIKE 1	0.025	3.294	Transcription factor regulating flower development and response to light stimulus.	[62]
DRB3 (DSRNA-BINDING PROTEIN 3)	0.020	2.984	Assists in miRNA-targeted RNA degradation.	[63]
SIGE (SIGMA FACTOR E)	0.032	2.808	Responds to effects of abiotic stresses. Phosphorylation of major sigma factor SIG1 in *Arabidopsis thaliana* inhibits the transcription of the *psaA* gene, which encodes photosystem-I (PS-I). This disturbs photosynthetic activity.	[64,65]
Reticulon family protein	0.029	2.772	Playing a role in promoting membrane curvature.	
Cytochrome c oxidase family protein	0.025	2.727	Essential for the assembly of functional cytochrome oxidase protein.	
DNA-binding S1FA family protein	0.049	2.717	Binds to the negative promoter element S1F.	
DNA photolyase	0.032	2.667	DNA repair enzyme.	
Zinc knuckle (CCHC-type) family protein	0.040	2.567	Zinc ion binding	
Monosaccaride transporter	0.025	2.547	Plays a role in long-distance sugar partitioning or sub-cellular sugar distribution.	
Nodulin MtN3 family protein	0.025	2.376	Key role in the establishment of symbiosis.	
Serine acetyltransferase, N-terminal	0.040	2.302	Catalyzes the formation of a cysteine precursor.	

**Table 4:** Top downregulated genes in DipC and TN.

Gene name	FDR	Fold Change	Gene Description	References
**Down-Regulated Genes in DipC**				
Dihydroxyacetone kinase	0.003	6.489	Glycerone kinase activity	
Phosphoglucomutase, putative/glucose phosphomutase, putative	0.007	6.471	Involved in controlling photosynthetic carbon flow and plays essential role starch synthesis. Down regulation of photosynthesis-related gene will lead to significant reduction in plant growth.	[66]
Auxin-induced protein 22D AUXX-IAA	0.003	4.627	Involved in stress defense response. Many AUXX-IAA genes were found to be down-regulated in Sorghum bicolor under drought conditions.	[67]
CP12-1, putative	0.014	4.390	Involved in calvin cycle, therefore linked to photosynthesis. Most drastic down-regulated genes which were photosynthesis-related was observed in barley (*Hordeum vulgare* L.)	[68]
PHS2 (ALPHA-GLUCAN PHOSPHORYLASE 2).	0.014	4.375	Encodes a cytosolic alpha-glucan phosphorylase.	
APRR5 (PSEUDO-RESPONSE REGULATOR 5), Pseudo ARR-B family	0.001	4.145	Linked to cytokinin-mediated regulation	
Thiamine biosynthesis family protein	0.002	4.132	Catalyses the activation of small proteins, such as ubiquitin or ubiquitin-like proteins.	
Zinc finger (C3HC4-type RING finger)	0.007	3.611	Mediate ubiquitin-conjugating enzyme (UBC-2) dependent ubiquitation.	[69]
WRKY40	0.033	3.104	Regulator of ABA signalling. It inhibits the expression of ABA-responsive genes ABF4, AB14, AB15, DREB1A, MYB2 and RAB18.	[70]
**Down-Regulated Genes in TN**				
AGL83 (AGAMOUS-LIKE 83)	0.025	4.374	DNA-binding transcription factor	
CRR23 (chlororespiratory reduction 23)	0.025	3.625	A subunit of the chloroplast NAD(P)H dehydrogenase complex, involved in PS-I cyclic electron transport. Located on the thylakoid membrane. Mutant has impaired NAD(P)H dehydrogenase activity. Part of dehydration repressing photosynthesis.	[71]
MYB30 (MYB DOMAIN PROTEIN 30)	0.032	3.250	Acts as a positive regulator of hypersensitive cell death and salicylic acid synthesis. Involved in the regulation of abscisic acid (ABA) signalling.	[72]
Photosystem II family protein, putative	0.029	3.158	Linked to photosynthesis. Down-regulation of photosynthesis-related genes during dehydration stress was observed in maize (*Zea mays*), which in turn leads to significant reduction in plant growth.	[73]
Phosphoesterase	0.047	3.136	Hydrolase activity, acting on ester bonds.	
Zing-finger (C3HC4-type)	0.045	2.947	Mediate ubiquitin-conjugating enzyme (UBC-2) dependent ubiquitation.	[69]
NHX2 (Sodium proton exchanger 2)	0.040	2.742	Involved in antiporter activity. Also involved in potassium ion homoeostasis and regulation of stomatal closure. Involved in the accumulation of K^+^ that drives the rapid stomatal opening. Down-regulation of genes related to stomatal regulation has been observed in soybean, which appears to be a part of dehydration response, leading to a reduction in the amount of stomata in leaves.	[74]
Inositol 1,3,4-trisphosphate 5/6-kinase	0.035	2.090	Part of IP3 signal transduction pathway.	[75]

**Table 5:** Overlapping up- and downregulated genes.

Gene Name	FDR	Fold Change	Gene Description	References
**Up-Regulated Genes**				
Beta-fructofuranosidase	8.90 × 10^−-4^	3.193	Catalyses the hydrolysis of sucrose. A rise in monosaccharide content caused by the Beta-fructofuranosidase can compensate for the decline in photosynthetic carbon assimilation indicated by the decrease in net photosynthesis.	[46,47]
**Down-Regulated Genes**				
MEE59 (maternal effect embryo arrest 59)	8.94 × 10^−-4^	8.580	Embryo development ending in seed dormancy.	
Calcineurin-like phosphoesterase family protein (CPPED1).	6.72 × 10^−-4^	5.857	Plays inhibitory role in glucose uptake. Down-regulation of CPPED1 improves glucose metabolism.	[48]
Putative lysine-specific demethylase JMJD5 Jumonji/Zinc-finger-class domain containing protein	0.003	4.971	Plays role in a histone demethylation mechanism that is conserved from yeast to human. Down-regulation may lead to an increase in methylated histones and hence general down-regulation of transcription.	[49]
MYB-like transcription factor	0.024	4.103	Arabidopsis homolog is known to regulate stomatal opening, flower development, and plays role in circadian rhythm.	[50]
F-box family protein (FBL14)	0.001	3.744	Functions in signal transduction and regulation of cell cycle.	
BRH1 (BRASSINOSTEROID-RESPONSIVE RING-H2)	0.007	2.899	BRH1 is known to influence stomatal density.	[51]
Bundle-sheath defective protein 2 family/bsd2 family	0.003	2.441	Protein required for post-translational regulation of Rubisco large subunit (rbcL).	[52]
Mitochondrial substrate carrier family protein	0.030	2.435	Involved in energy transfer.	

FDR: false discovery rate.

**Table 6:** Vertex degrees of differentially expressed transcription factors.

DipC				TN			
Probe-Set	Name	V°Whole	V°Drought	Probe-Set	Name	V°Whole	V°Drought
Gma.16733.1.S1_at	WRKY40	68	17	GmaAffx.45249.1.S1_at	CONSTANS-like 1	16	3
Gma.6670.1.S1_at	PRR7	49	7	GmaAffx.84566.2.S1_at	MYB60	8	3
GmaAffx.33796.3.S1_at	Zinc-finger like C2H2	45	7	GmaAffx.86517.1.S1_at	AGL83	6	1
GmaAffx.92679.1.S1_s_at	ATAUX2-11	41	9	Gma.1576.1.S1_at	Zinc-finger C3HC4	5	1
GmaAffx.35309.1.S1_s_at	GRF2	35	6				
GmaAffx.65059.1.S1_at	bHLH	32	7				
GmaAffx.90399.1.S1_at	C3HC4 Zinc-finger	31	9				
Gma.15774.1.S1_at	Zinc-finger C3HC4	26	3				
GmaAffx.53180.1.S1_at	PRR7	25	9				
GmaAffx.80492.1.S1_at	PRR5	9	2				
GmaAffx.73009.2.S1_at	WRKY51	7	5				
**Common TFs**							
GmaAffx.60283.1.S1_at	BRH1	42	6				
GmaAffx.9286.1.S1_s_at	MYB	27	4				
Gma.17248.1.A1_at	JMJD5	26	3				
GmaAffx.10162.1.S1_at	MEE59	13	3				

V° refers to the number of links of each transcription factor (TF) node, in either the whole genotype-specific network, or merged dehydration-specific network.

## Supplementary Materials

The following are available online at www.mdpi.com/2073-4425/8/4/121/s1. Figure S1: Treatment Regimes, Figure S2: Genotype specific 3D PCA scatter plots, Figure S3: Ranked mean intensities of water-sufficient genotype samples, Figure S4: Categories of system response in gene expression, Figure S5: Comparison between bambara groundnut and soybean for expression levels for dehydration-associated genes under water-sufficient conditions. Table S1: Dehydration-associated genes expressed under water-sufficient condition in DipC, Table S2: Dehydration-associated genes expressed under water-sufficient condition in TN, Table S3: Dehydration-associated genes expressed under water-sufficient condition in both DipC and TN, Table S4: Comparison of water-sufficient conditions transcriptomes of DipC and TN, Table S5: Upregulated DipC genes as a result of water-limited treatment (*p*-value <= 0.05, Abs. F.C > 2), Table S6: Downregulated DipC genes as a result of water-limited treatment (*p*-value <= 0.05, Abs. F.C > 2), Table S7: Upregulated TN genes as a result of water-limited treatment (*p*-value <= 0.05, Abs. F.C > 2), Table S8: Downregulated TN genes as a result of water-limited treatment (*p*-value <= 0.05, Abs. F.C > 2), Table S9: List of differentially expressed genes (water-limited versus water-sufficient) common to DipC and TN when sample D.DipC.rep2 is excluded from the analysis, Table S10: GO-term overrepresentation of all the gene-sets in Soybean GeneChip array to compare DipC, TN, and Soybean datasets, Table S11: DipC Co-expression network, Table S12: TN Co-expression network.
